# Larry Jacoby: An appreciation of his early research

**DOI:** 10.3758/s13421-026-01927-w

**Published:** 2026-08-26

**Authors:** Henry L. Roediger

**Affiliations:** Department of Psychological and Brain Sciences, Washington University in St. Louis, One Brookings Drive, Box 1125, St. Louis, MO 63124 USA

**Keywords:** Two-store memory models, Dissociations among memory measures, Priming, Implicit memory, Explicit memory

## Abstract

Larry Jacoby was one of the most creative experimental psychologists of his generation. I describe four papers from early in his career that were influential to debates at the time and that are still well worth reading today. These are Jacoby and Bartz (*Journal of Verbal Learning and Verbal Behavior, 11*[4], 561–565, 1972), Jacoby ((*Journal of Verbal Learning and Verbal Behavior, 17*[6], 649–667, 1978), Jacoby and Dallas (*Journal of Experimental Psychology: General, 110*[3], 306–340, 1981), and Jacoby (*Journal of Verbal Learning and Verbal Behavior, 22*[5], 485–508, 1983). The last two papers showed that dissociations among memory measures, analogous to those observed in amnesic patients (relative to controls), could be obtained in college students. These are just a selection of his famous findings, which include how people can become famous overnight (Jacoby et al., *Journal of Personality and Social Psychology, 56*[3], 326–338, 1989), how to deceive the elderly (Jacoby, *Cognitive Neuropsychology, 16*(3/5), 417–436, 1999), and, of course, the process dissociation procedure (Jacoby, *Journal of Memory and Language, 30*[5], 513–541, 1991). Jacoby’s influence on cognitive psychology and related fields will endure.

I first heard Larry Jacoby’s name when Endel Tulving walked into a seminar room at Yale in the fall of 1971 and asked, “Does anyone know a man named Larry Jacoby?” As he expected, no hands went up. At the time, Endel was a professor at Yale and the editor of the *Journal of Verbal Learning and Verbal Behavior* (later renamed the *Journal of Memory and Language*). The meeting was called “Memory Lunch,” and I was a graduate student in attendance. Endel waved a paper he was carrying and said, “I have received a fine paper by Jacoby. He is young, just starting out, and I predict he has a great future in our field.” That statement is close to a direct quote, and the prediction was certainly borne out.

I met Larry in the fall of 1973 when I gave a talk at Ebbinghaus Empire at the University of Toronto. I had recently taken a position at Purdue University. That year, Larry was visiting Erindale College, part of the University of Toronto, working with Gus Craik, and he came over for my talk at the main campus and to visit with me and others. We met in a small conference room, and Larry asked if I would mind if he smoked. I was a nonsmoker, but following the custom of the times, I said no. Larry remarked, “It’s a nasty habit. I need to quit soon.” He never did, though he tried many times. When we were colleagues at Washington University, he told me more than once, “I can’t think unless I smoke.”

My paper commemorating Larry will focus on his early work, from 1970 to 1983, since I expect many other writers will focus on his more recent research. I begin with the Jacoby and Bartz paper, which Tulving admired and published in *JVLVB,* then describe three others. I selected only a few papers due to space constraints, and it was difficult. Larry was one of our field’s most clever experimentalists, and his contributions are numerous. As of this writing, ten of his papers have been cited more than 1000 times. I have necessarily reported these studies in abbreviated form and taken a few shortcuts. I encourage readers to examine the papers in depth.

## Jacoby and Bartz (1972)

In the early 1970s, several models postulated separate short-term and long-term memory stores, with the attendant idea that transfer from short-term to long-term store was accomplished by rehearsal (Glanzer & Cunitz, 1966; Waugh & Norman, 1965) or via other possible mechanisms^1^ (Atkinson & Shiffrin, 1968). In these models, rehearsal serves two functions: maintaining information in the short-term store and transferring it to the long-term store. One piece of evidence supporting these functions of rehearsal was the discovery of the negative recency effect first reported by Craik (1970). He presented subjects with ten lists of 15 words for initial recall on each day for 4 days. After all lists had been studied and immediately recalled on each day, subjects were given a final free recall test in which they recalled as many words as possible from all lists. Craik found the usual strong recency effect over the last few items in initial recall (a positive recency effect), but in final free recall, he observed a negative recency effect: Recall of the last few items was worse than recall in the rest of the list despite their being well recalled earlier (see Craik, 1970, Fig. 2). The two-store model accounted for negative recency by assuming that the last few items in the list were rehearsed less than earlier items.

Jacoby and Bartz (1972) proposed that rehearsal served only one function: maintenance in the short-term store, with no transfer to LTS via this mechanism. They tested this prediction using a straightforward between-subjects design, with ten 20-word lists presented at a rate of 2 s/word across all conditions. In one condition, subjects heard lists of 20 words and recalled each list immediately after hearing it, the standard method of single-trial free recall. In a second condition, subjects were presented with the lists in five-word blocks, with a 15-s unfilled delay period after each block; this procedure allowed extra rehearsal after each five-word block and should eliminate the negative recency effect and boost overall recall. Initial recall occurred after the unfilled delay period for each list. In the third condition, subjects were again given the 20 words in five-word blocks, but now the periods between blocks were filled with mental arithmetic to prevent rehearsal. Again, initial list recall occurred after the filled delay periods. At the end of the experiment, subjects in all conditions received an untimed final free recall over all 200 presented words. The prediction is clear from the vantage of two-store models with maintenance rehearsal as the mechanism of transfer between stores—final recall should be best for the condition with unfilled delays, with perhaps no difference in recall between the continuous presentation-immediate recall condition and the condition with filled delays after blocks of words.

In the initial recall conditions, overall recall was superior in the unfilled delay condition (48%) relative to the filled delay condition (37%) or the immediate recall condition (32%). Recall of the last five items was about the same in the unfilled delay and immediate recall conditions, but much poorer in the filled delay condition. These results are as expected.

In final free recall, the primary interest is in the final five items presented. Contrary to expectations from the two-store models, recall in the filled-delay condition was superior to that in the unfilled-delay condition, despite greater opportunities for rehearsal in the latter condition. Rehearsal did not seem to be the process (or not the sole process) causing transfer from STM to LTM.

Why should final recall have been greater for the condition with filled delays relative to that with unfilled delays between five-word blocks on the final block of items? Jacoby and Bartz (1972) suggested that when subjects anticipated a filled delay, they engaged in a different type of processing to store and retrieve words relative to when a short delay was expected: “Tulving (1968) has suggested that items recalled immediately after presentation differ from those recalled after a delay in terms of the type of retrieval cues employed. If this distinction is accepted, initial recall after a filled delay might employ retrieval cues that will also be available at the time of final free recall. That is, retrieval from LTM might also be more effective than a retrieval from STM as a means of increasing final free recall performance” (p. 564). Another modification of this last statement, using more contemporary language, is that the effect of retrieval from LTM leads to a testing (or retrieval practice) effect, whereas a retrieval from STM does not, a proposition for which there is ample support (e.g., Karpicke & Roediger, 2007; Landauer & Bjork, 1978).

The primary finding of Jacoby and Bartz’s paper—that maintenance rehearsal is not responsible for transferring information to LTM—has been confirmed by much later work (e.g., Craik & Watkins, 1973), although some reports indicate that rehearsal may benefit long-term recognition but not recall (e.g., Glenberg, 1977; Woodward et al., 1973; see Greene, 1987, for an early review).

## Jacoby (1978)

**Fig. 1:**
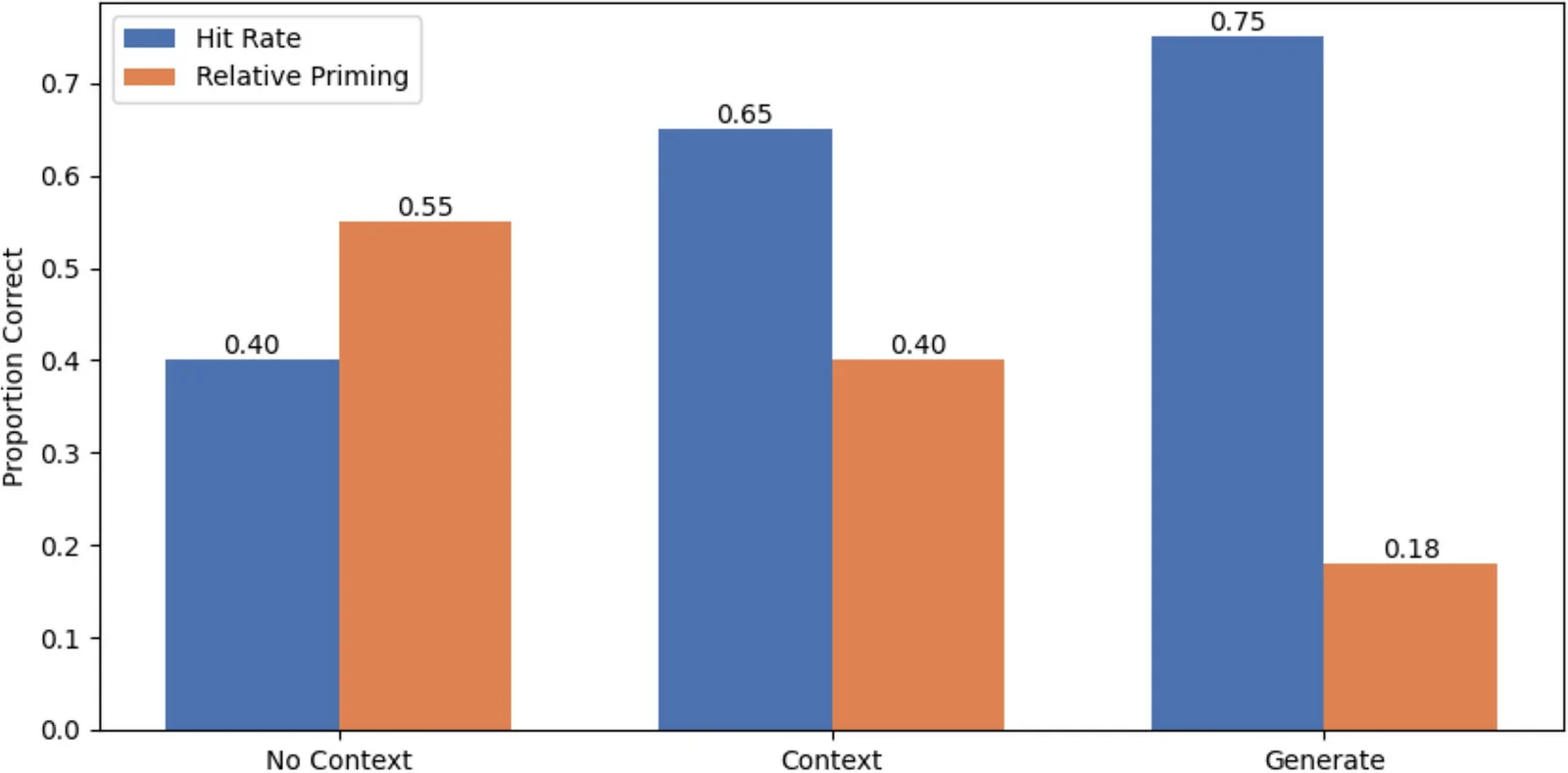
Results from Jacoby (1983, Experiments 2 and 2R). Probability correct is on the abscissa. The blue bars show recognition data (hit rate) and the orange bars show relative priming data on the word identification test (see the text for explanation). Although the subjects are treated the same until the time of the test, the two retention measures show opposite patterns as a function of the independent variable. The figures are adapted from the data in Jacoby (1983, Table 2). (Color figure online)

Two papers published in 1978 demonstrated a generation effect. The most cited one, perhaps because it named the effect, was by Slamecka and Graf (1978). However, Jacoby (1978) also obtained a generation effect in an experiment in which he compared reading words with solving a simple problem to construct the word. Compared with the procedure by Slamecka and Graf, which used materials like *Opposites: open–????* to cause subjects to generate a response, Jacoby had subjects either generate words from a semantic clue and a pattern of letters (e.g., foot–sh--) or read the pair aloud (*foot–shoe*) in a within-subjects design. Despite the simplicity of the generation task, he observed a significant effect on later recall; in one experiment, cued recall of the target words was .26 in the Read condition and .57 in the Construct or Generate condition, reflecting a notable 31 percentage point improvement from just omitting two letters. (Data were estimated from Fig. 1 on page 653 of his paper.)

Jacoby (1978) also included a manipulation of spacing, such that pairs were read twice in a massed or spaced fashion or read and then constructed (massed or spaced). When constructing a word had been immediately preceded by reading it, the benefit of construction was eliminated; even though the pair was presented twice, recall was lower (.47) than when a pair was presented only once in the Construct condition (.57). Jacoby argued that the process of generation or construction was short-circuited or “trivialized” when subjects had just read the word. However, when the Read-Construct condition was given under spaced conditions, recall was .78, for a 31 percentage point spacing effect. The spacing effect in the Read-Read condition was only 7%, although still significant.

Jacoby (1978) interpreted the beneficial effects of construction or generation as possibly involving more effort, creating a more distinctive encoding. In addition, he suggested that construction may engender more arousal than reading. He also discussed the implications of his results for theories of the spacing effect. My focus here is on the powerful effect of construction or generation on later recall.

## Jacoby and Dallas (1981)

Craik and Lockhart (1972) introduced their influential levels-of-processing framework for memory research, which guided the field for years to come and still does. They reviewed many prior papers that presented evidence for the benefit of semantic processing of words over other types of processing involving words’ physical characteristics (e.g., Hyde & Jenkins, 1969; Schulman, 1971). A few years later, Craik and Tulving (1975) reported ten experiments using a paradigm that instantiated three levels of processing of words. They asked students questions that elicited orthographic processing (e.g., *stair*—does the word contain an *a*?), phonemic processing (does the word rhyme with bear?), or semantic processing (is the word something found in a house?). Half the questions required a *yes* answer, as in these examples, and half required a *no* answer. Across the ten experiments, Craik and Tulving showed that semantic processing led to greater recognition than phonemic processing, which in turn led to greater recognition than orthographic processing. This confirmed the levels-of-processing effect: deeper processing (semantic) led to better recognition than shallower levels (phonemic, then orthographic). Further, items requiring *yes* answers led to greater recognition than those requiring *no* answers, at least for the deeper levels of processing.

In the first in a series of six excellent experiments, Jacoby and Dallas (1981) employed the Craik and Tulving (1975) paradigm, but they asked what effect the manipulation would have on an indirect measure of retention, viz., word identification—how well students could identify briefly presented words (~35 ms) when they had been previously presented relative to identifying words that had not been presented. The authors expected this perceptual identification task to reveal the effects of prior experience of words—that is, priming—relative to control words that had not been presented in the experimental context. The difference in proportion of correct identifications between the studied and nonstudied words indicated priming on the test by prior study. Jacoby and Dallas also used a standard recognition test with words shown clearly at a slow rate after presentation of the same lists; recognition in some experiments occurred after word identification, but in others it occurred after presentation of the lists.

Following the standard manipulation (3 levels of processing × 2 answers), Jacoby and Dallas (1981) reported the standard levels-of-processing effect on recognition, with semantic processing yielding the greatest recognition and prior *yes* responses yielding better recognition than *no* responses. However, on the indirect perceptual measure, they showed priming for studied words (relative to a nonstudied baseline) in word identification, indicating that the test measures memory. Surprisingly, they found no levels-of-processing effect with this measure and no difference in priming between items answered *yes* and *no* during study. See Table 1 for both the recognition hit rates and the priming results. I report both the standard priming and relative priming results. The standard priming measure is the difference between the proportion of words identified in the study conditions and the base rate (proportion of words identified without prior presentation). Because the base rate is so high (.65), the relative priming value is also of interest. This is calculated by dividing the standard priming value by .35 (1.00 - .65), the maximum amount of priming possible under these conditions. Although this represents a linear transformation of the data, it shows the magnitude of priming unconstrained by the high base rate.

**Table 1:** Results from Jacoby and Dallas (1981, Experiment 1)

Question Type						
	Physical		Rhyme		Semantic	
	Yes	No	Yes	No	Yes	No
Recognition Hit Rate	.51	.49	.72	.54	.95	.78
Priming Word Identification	.13	.16	.17	.15	.15	.18
Relative Priming	.37	.45	.48	.43	.43	.51

The six conditions are listed at the top, and the rows report recognition results (hit rate) in the top line and two measures of priming on the word identification test in the next two rows. The difference between priming and relative priming measures is provided in the text. The table is adapted from Jacoby and Dallas (1981), Table 2

These results have been replicated using similar perceptual priming measures such as completing fragmented words such a _ _ a _ _ i n for assassin (e.g., Roediger et al., 1992). In five additional experiments Jacoby and Dallas (1981) examined other variables on both standard recognition tests and priming in the word identification task, sometimes finding parallel effects for the two variables. For example, spacing of repetitions enhanced both recognition and priming (Experiment 5).

I first heard Larry report these results in an invited talk at the Midwestern Psychological Association meetings in 1981. I recall being surprised, because the “levels” effect was so robust and I often used it as a reliable classroom demonstration experiment with recognition testing. The data in Table 1 show the recognition hit rate increasing from about .50 in the physical condition to .95 in the semantic-yes condition, but with no discernible effect at all in priming on the word identification test. What was so different about this new measure? It clearly reflected learning and thus memory, because of the robust priming observed. But it was a different kind of memory, later called implicit memory by Graf and Schacter (1985; see Schacter, 1987, for a historical review) or an indirect measure of memory (Jacoby, 1984).^2^

Jacoby and Dallas (1981) argued that their results could be interpreted in several ways. For example, “The results of the experiments reported earlier can be described in terms of the semantic versus episodic memory distinction proposed by Tulving (1972). Presentation of an item during study may activate the semantic memory representation of a word without a new episodic trace being formed. Recognition memory, an episodic memory task, may depend on the formation of an episodic trace. In contrast, perceptual recognition or word identification, a semantic memory task, depends only on the level of activation of the semantic representation of the tested item. Variables such as level of processing, then, can be described as influencing the probability of an episodic trace being formed, thereby affecting recognition memory but not priming on the word identification test. Parallel effects in recognition memory and priming may simply show that differences in semantic memory can influence performance of an episodic memory task” (p. 335). They also appealed to the two bases of recognition proposed by Atkinson and Juola (1974) and Mandler (1980). In these theories, one basis of recognition is relatively automatic and relies on processing fluency. The other process is under greater cognitive control and involves a search of (episodic) memory. Jacoby’s (1983) experiments, discussed next, led him to revise his theoretical interpretation. However, in later papers, Jacoby emphasized automatic and controlled processing in memory tasks and developed the clever idea of pitting these processes against one another to determine the relative amounts of each involved in a memory task (Jacoby, 1991).

## Jacoby (1983)

The experiments in this 1983 paper combined aspects of the two prior publications covered here: Effects of generating versus reading (in Jacoby, 1978) as measured by both recognition memory and word identification tests (Jacoby & Dallas, 1981). Jacoby (1983) distinguished between data-driven and conceptually-driven processes as applied to episodic recognition and word identification. Consider saying a word aloud from different clues to the nature of the word. If you see the word *cold* on a computer screen and produce it, this is accomplished by data-driven processing: The word is processed through the perceptual system via bottom-up processing; there is no other way. On the other hand, if you see the *opposite of hot*–*????,* and you produce *cold*, this occurs via top-down, conceptual processing. The “data” that represent the word are not shown at all. A third possibility combines conceptual and perceptual processing, in which “*Opposite of hot–cold*” is displayed on a screen. Producing the word would be accomplished via a combination of top-down and bottom-up processing in this case. Jacoby used the term conceptually driven to refer to top-down processing and data-driven processing to refer to bottom-up processing. Importantly, he further proposed that episodic recognition drew primarily on conceptual processes, whereas word identification relied on data-driven processing.

Jacoby’s (1983) experiments tested these ideas. In the first three experiments, he used the three means of producing a word outlined in the previous paragraph as the study or encoding conditions, viz., No Context, Context, and Generate conditions. During a first phase, subjects said aloud a word presented in the No Context (xxx–COLD), Context (hot–COLD), or Generate (hot–????) conditions, in which the two elements were presented successively on a computer screen. All the target words were antonyms that could be readily generated from their opposite. Following encoding of many words in each of these conditions (within subjects), students were then given either a standard yes/no recognition test in which studied words were presented one at a time or a word identification test in which the same words were presented quite rapidly. In the recognition test, subjects indicated whether each item was previously presented in the list by providing a yes/no response. In the word identification test, subjects were presented with the same words at a fast rate determined by prior testing for each subject (averaging about 35 ms/word). The subject’s task was to report the word, if possible.

Based on prior research on the generation effect and related phenomena, Jacoby proposed that recognition was a conceptual (meaning-based) test, because generating words under these conditions leads to greater recognition than reading them (Slamecka & Graf, 1978). However, he proposed that word identification from brief glimpses was a data-driven test; this supposition was also supported by prior research (Jacoby & Dallas, 1981; Winnick & Daniel, 1970). If this were so, then presentation of words out of context should lead to the greatest priming and the least priming should occur in the generate condition (where the “data” are never seen), with the context condition intermediate.

In short, Jacoby’s (1983) first three experiments conformed to a 3 (encoding conditions) × 2 (test conditions) design, with the independent (encoding) variable manipulated within-subjects and the dependent (test type) variable manipulated between subjects (in the data presented here). Further, Jacoby predicted that manipulation of the encoding variable would have opposite effects on the two tests.

The results from Jacoby (1983, Experiment 2) show that his predictions were borne out. The recognition results (blue bars in Figure 1) replicated those of others, showing that generating a word from a conceptual clue enhanced recognition relative to reading it without context. Reading the word in context produced an intermediate level of recognition. In Jacoby’s (1983) terms, the greater conceptual processing engendered at encoding, the higher the level of performance on a test that requires conceptual (semantic) processing.

On the other hand, priming on the word identification test produced the opposite pattern of performance. The measure in the figure is relative priming. Baseline performance (the proportion of identified words that were new) was .60. The identification proportions in the three studied conditions were .82, .75, and .67 for the No Context, Context, and Generate conditions yielding priming above the baseline of .22, .15, and .07, respectively. I divided each of these by .40 (1.00 −.60) to provide a relative priming value, or priming relative to the amount of priming that is possible to observe given the baseline values. (Of course, these numbers are a linear transformation of the absolute priming scores.) As shown by the orange bars in Fig. 1, priming was greatest in the No Context condition, intermediate in the Context condition, and smallest in the Generate condition. Because the word identification test requires seeing the data, prior presentation of the word by itself produced the greatest priming. When the word was placed in context, priming declined, presumably because top-down, conceptually driven processing undermined some data-driven processing. In the Generate condition, when the word was generated and not seen at all, the least priming occurred.

Jacoby’s (1983) first three experiments yielded opposing patterns of results on two memory-based tests, one direct (explicit) and the other indirect (implicit). Jacoby argued against interpreting the results as reflecting differences in memory systems, such as recognition reflecting episodic memory and priming reflecting the expression of semantic memory. Rather, he wrote that “perceptual identification and recognition memory utilize different forms of information rather than reflecting the operation of different memory systems” (Jacoby, 1983, p. 500). This striking pattern was conceptually replicated by Blaxton (1989) and Srinivas and Roediger (1991), among others, although see Masson and MacLeod (1992). The pattern shown in Fig. 1 represents a crossover interaction: Manipulating the same variable affected the two memory tests differently. This interaction (or dissociation) between tests provides strong evidence that different processes are at work. Many other experiments show that a variable affects one test (usually recall or recognition) but not the other (usually an implicit test like word priming), as in Jacoby and Dallas’s (1981) experiments.

Jacoby’s (1983) interpretation of his results aligns with the logic of transfer-appropriate processing, although Jacoby never used that phrase in his paper. The phrase was introduced in an important paper by Morris et al. (1977) and refers to the idea that the type of processing afforded to material during learning determines which type of memory test should produce superior performance during retrieval. They argued that no type of encoding is inherently superior to any other (e.g., semantic relative to phonemic, as in the levels of processing framework), but the outcome depends on the type of test used. To support their argument, they conducted a levels-of-processing experiment with two encoding conditions and two explicit tests. They presented sentences such as “The **eagle** took flight” with instructions in one condition to remember the boldfaced words, or, in the other, to indicate (yes or no) whether the word rhymed with *beagle* (with no instructions about remembering the words or a memory test). These conditions instantiated semantic or phonemic processing. One test was a standard yes/no recognition test in which old words were mixed with new ones. The other test was a rhyme recognition test in which a list of words was presented, none of which had been seen before, and the subjects’ task was to indicate whether or not each word on the test rhymed with a studied word. Thus, in our example, the target word on the test might be *regal*.

Morris et al. (1977) found that on the standard recognition test, semantic processing led to better recognition than phonemic processing. However, on the rhyme recognition test, prior phonemic processing led to better recognition than prior semantic processing, a reversal of the standard levels-of-processing effect. (This result occurred only for studied items that required a *yes* response during study.)^3^ The opposite results of encoding manipulations on performance on two different tests are like those in Jacoby (1983).

Jacoby’s (1983) results could be interpreted in two ways. He preferred an interpretation in terms of the type of information—conceptual or perceptual (data-driven) processing—that led to better transfer on tests that required the same type of processing during study. Tests that matched study–test processing led to greater performance than tests that mismatched study conditions. However, the same results could just as easily be interpreted as reflecting the operation of different memory systems, episodic and semantic. With only two tests—one explicit and conceptually driven and the other implicit and data-driven, the two explanations can serve equally well.

In the early 1980s, I collaborated with Paul Kolers on a paper (invited by Fergus Craik for the *Journal of Memory and Language*) intended to bring Kolers’ research on reading inverted text to the attention of memory researchers (Kolers & Roediger, 1984). Kolers (e.g., 1973; Kolers & Perkins, 1975) showed that subjects trained to read various text inversions exhibited specificity in reading sentences inverted in the same manner on a test, even when the sentences were novel. In addition, when they read the same passages inverted in the same way, they showed savings even a year later, despite being unable to explicitly recognize the passages they had read from ones they had not read (Kolers, 1976). Kolers interpreted his work as supporting an approach he called procedural processing, which was like transfer-appropriate processing in that it emphasized matching encoding and test procedures to maximize performance. (It would take me too far afield to describe Kolers’ work in more detail.) At the same time, I was having a friendly argument with Endel Tulving and others over whether one needed to postulate memory systems to explain differences (dissociations) obtained on memory tests or whether a different, more straightforward explanation would work (e.g., Roediger, 1984). My collaboration with Kolers and the publication of Jacoby’s (1983) results led me to develop a transfer-appropriate processing account of differences between explicit and implicit memory tests that seemed to undercut the foundations of using explicit/implicit tests to support different memory systems.

My students and I argued that tests, whether explicit (episodic) or implicit (semantic), could be perceptually or conceptually based, and thus there was no necessary correlation between explicit memory and conceptual tests or between implicit memory and perceptual tests, even though nearly all research in the literature used this comparison. We argued that the critical evidence needed to distinguish between the memory systems and transfer-appropriate processing (or procedures) accounts was to unconfound the correlation in the literature between explicit and implicit tests with their conceptual and perceptual nature. What was needed was to vary, in the same experiment, two implicit tests and two explicit tests, with one in each case requiring perceptual processing and the other conceptual processing (Roediger & Blaxton, 1987; Roediger et al., 1989).

My students and I conducted many experiments along these lines. For example, we showed that two perceptual implicit memory tests could be dissociated (Weldon & Roediger, 1987), and a later chapter reviewed experiments demonstrating dissociations among implicit memory tests (Roediger et al., 1989). Blaxton (1989), in her dissertation, reported three compelling experiments in which explicit (episodic) and implicit (semantic) tests were crossed with tests requiring conceptual or perceptual processing. For example, in Experiment 2, she manipulated the modality (auditory or visual) in which words were presented in a list, because prior research had shown that modality had a large effect on perceptual implicit tests but not on conceptual explicit tests. In addition, she used two explicit tests, either free recall (episodic, conceptual) or a new test she developed called graphemic cued recall (also episodic but with perceptual retrieval cues). In this test, the retrieval cues were similar in appearance and sound to the studied word, and subjects were told to ignore the cue’s meaning and use its appearance and sound to retrieve a studied word. For example, if *hemlock* were one of the studied words, subjects would be given a word like *hamhock* as a cue. The test is episodic, because students were told to try to retrieve words from the studied list that looked or sounded like the cue word. Similarly, two implicit tests (putatively testing semantic memory) were used, and the list presented was intended to prime some answers but not others. The perceptual or data-driven test was word fragment completion, so students may have been given h _ m _ o _ c k. The other, semantic, test involved answering general knowledge questions, so subjects were given the question “What did Socrates drink at his execution?” This is a conceptual/semantic test. In the case of both implicit tests, students were told to answer as well as possible, with no mention made of the list that had been presented earlier under incidental learning conditions (typical implicit test conditions).

Blaxton’s (1989) results were clear-cut. On the two conceptually driven tests (free recall and answering general knowledge questions), no effect of modality was observed. However, on the two perceptual tests (graphemic cued recall and word fragment completion), large effects were observed, with visual presentation yielding better performance than auditory presentation in both cases. Thus, two episodic memory tests were dissociated (free recall and graphemic cued recall), and two semantic tests were dissociated (word fragment completion and answering general knowledge questions). The variable controlling the dissociations was the perceptual or conceptual nature of the tests. In sum, the best account of the dissociations between tests was the distinction between conceptual and perceptual types of processing. Whether the tests were episodic or semantic did not affect the results.

Of course, it is always possible to account for contrary results by changing one’s theory. When Tulving et al. (1982) introduced the word fragment completion test, they must have worried whether it reflected semantic memory, because at the end of the article they wrote: “Finally, it is worth noting that although the priming effects discussed in this article were demonstrated in what is clearly a semantic memory task, it is not clear that they can be regarded as a phenomenon of semantic memory.” They ended by writing that “we are tempted to think that they reflect the operation of some other, as yet little understood, memory system” (both quotes from page 341). Of course, discordant data might be accommodated within the memory-systems perspective by introducing a new memory system after the fact. Following research clearly showing dissociations between episodic and semantic memory tasks that fell along the perceptual/conceptual contrast, Tulving and Schacter (1990) argued for a new memory system, the perceptual representation system, which incorporated perceptual processing into the memory-systems view. Of course, this system was hardly “little understood,” as Tulving et al. (1982) had suggested. As Larry Jacoby commented to me at the time, “Tulving and Schacter have discovered the perceptual system.”

It is a bit surprising that Tulving resisted the ideas of transfer-appropriate processing and related ideas, because they can be considered a broadening of his encoding specificity principle (Tulving & Thomson, 1973), which emphasizes the match between the encoded features of a memory and the features of the retrieval cues at the time of test. Although Tulving emphasized a match of features (e.g., Flexser & Tulving, 1978) and others emphasized a match of processing (Fisher & Craik, 1977; Jacoby, 1983; Kolers, 1973; Morris et al., 1977), the spirit of the ideas is highly similar. Tulving emphasized that the match in features between encoding and retrieval determined memory performance, and he reported and reviewed many such instances between recall and recognition (Tulving, 1983, Chapters 10–13), both episodic memory tests. Of course, it has long been known that explicit measures of retention, such as recall and recognition, can be dissociated (see Tulving, 1972, for an early review). The encoding specificity principle predicts as much (see Roediger & Tekin, 2025, for a recent review).

## Conclusion

Larry Jacoby was a master experimentalist. In this brief encomium, I have provided four examples from his early research. The contributions reported here are necessarily selective but highly influential; the last three have each been cited more than 1,000 times as of this writing, and the Jacoby and Dallas (1981) paper has been cited more than 4,000 times. These papers reported results that mirrored the dissociations observed in amnesic patients, as Jacoby and Witherspoon (1982) emphasized in another paper cited over a thousand times. I was tempted to extend my range to 1989, because he had three papers and a chapter, all published that year, each cited more than 1,000 times. Larry Jacoby was a great psychologist and an interesting and complex man. His contributions to cognitive psychology and other fields will be influential for many years to come.

My intellectual debt to Jacoby should be clear from this article, but it runs much deeper than is indicated here. I followed his work carefully from the early 1970s until his retirement in 2015. We were colleagues at Washington University from 2004 until his retirement, and I marveled at how he continued to design novel experiments that yielded important insights. He has trained a cadre of students who have followed in his footsteps, making their own important contributions. Larry and I had many delightful conversations at professional meetings in our early days and, much more often, after we became colleagues. We never managed to collaborate much. We are co-authors on two papers, and I also have one with his son, Derek, who was an undergraduate at Rice University while I taught there (Roediger et al., 1996). I feel confident that Larry Jacoby’s work and influence will long endure.

## Footnotes

1 One reviewer questioned the claim that the latter model provided for other processes besides maintenance rehearsal. Atkinson and Shiffrin (1968) argued that there were two purposes of rehearsal, one of which was maintenance. They went on to say that “rehearsal serves the purpose of increasing the strength built up in a long-term store, both by increasing the length of stay in STS (during which time a trace is built up in LTS) and by giving coding and other storage processes time to operate. Indeed, almost any kind of operation on an array of information (such as coding) can be viewed as a form of rehearsal, but this paper reserves the term only for the duration-lengthening repetition process.” That statement leaves their claim somewhat ambiguous, but they also wrote: “Certainly if the subject is engaged in coding or other active transfer strategies, the time spent in attending to an item should be directly related to the amount of transfer to LTS.” Thus, they also allow for what later came to be called elaborative rehearsal. In making this point, Wixted (2024) commented that “Atkinson and Shiffrin (1968) chose to mainly focus on the ‘duration-lengthening repetition process’ of verbal information instead of manipulating coding processes. A ‘repetition process’ sounds like the veritable definition of maintenance rehearsal, so perhaps this is why their model is often incorrectly viewed as a model of the effects of maintenance rehearsal, per se.” The quotes are from Wixted’s (2024) paper, page 3.

2 As a reviewer pointed out, I should not have been so surprised by these findings because three reports existed showing that even explicit memory tests could fail to reveal a levels-of-processing effect if the retrieval cues were phonemic or orthographic in nature rather than the semantic cues typically used on explicit tests (Fisher & Craik, 1977; McDaniel et al., 1978; Morris et al., 1977).

3 Jacoby (1975) reported a pattern of results similar to those of Morris et al. (1978).

## Declarations

The first submission of this manuscript was rejected automatically because I forgot to add declarations about the critical matters listed below. That task has now been completed.

### Availability of data and materials

Only aggregated data are provided in Jacoby’s papers. Neither he nor I can provide raw data. Jacoby is dead, and I am old.

### Code availability

If Jacoby and his lab had a secret code, I’m afraid I don’t know it. Maybe Andy Yonelinas does.
